# Growth Rate as a Direct Regulator of the Start Network to Set Cell Size

**DOI:** 10.3389/fcell.2017.00057

**Published:** 2017-05-26

**Authors:** Martí Aldea, Kirsten Jenkins, Attila Csikász-Nagy

**Affiliations:** ^1^Molecular Biology Institute of Barcelona, Consejo Superior de Investigaciones CientíficasBarcelona, Spain; ^2^Departament de Ciències Bàsiques, Universitat Internacional de CatalunyaBarcelona, Spain; ^3^Randall Division of Cell and Molecular Biophysics and Institute of Mathematical and Molecular Biomedicine, King's College LondonLondon, United Kingdom; ^4^Faculty of Information Technology and Bionics, Pázmány Péter Catholic UniversityBudapest, Hungary

**Keywords:** cell size, cell cycle, size control, budding yeast, mathematical model

## Abstract

Cells are able to adjust their growth and size to external inputs to comply with specific fates and developmental programs. Molecular pathways controlling growth also have an enormous impact in cell size, and bacteria, yeast, or epithelial cells modify their size as a function of growth rate. This universal feature suggests that growth (mass) and proliferation (cell number) rates are subject to general coordinating mechanisms. However, the underlying molecular connections are still a matter of debate. Here we review the current ideas on growth and cell size control, and focus on the possible mechanisms that could link the biosynthetic machinery to the Start network in budding yeast. In particular, we discuss the role of molecular chaperones in a competition framework to explain cell size control by growth at the individual cell level.

## Brief introduction

Size and shape have important consequences in cell physiology, and cells are thought to adjust their physical dimensions in order to optimize functionality in a diverse world of developmental options and environmental conditions. Many intrinsic and extrinsic factors have determinant effects on cell size through molecular pathways that have been uncovered mostly in the last two decades, and there are excellent reviews covering all aspects of cell size control from key molecules to physiological implications (Jorgensen and Tyers, 2004; Cook and Tyers, 2007; Marshall et al., 2012; Navarro et al., 2012; Turner et al., 2012; Lloyd, 2013; Schmoller and Skotheim, 2015; Wood and Nurse, 2015). Here, we will focus on a universal trait of cell size control: the faster cells grow, the larger they are. While this observation was made almost a century ago, the precise molecular mechanisms that specifically act to coordinate growth and proliferation, so as to set cell size as a function of growth rate, are just beginning to emerge.

## Cell size depends on growth rate

**Figure 1:**
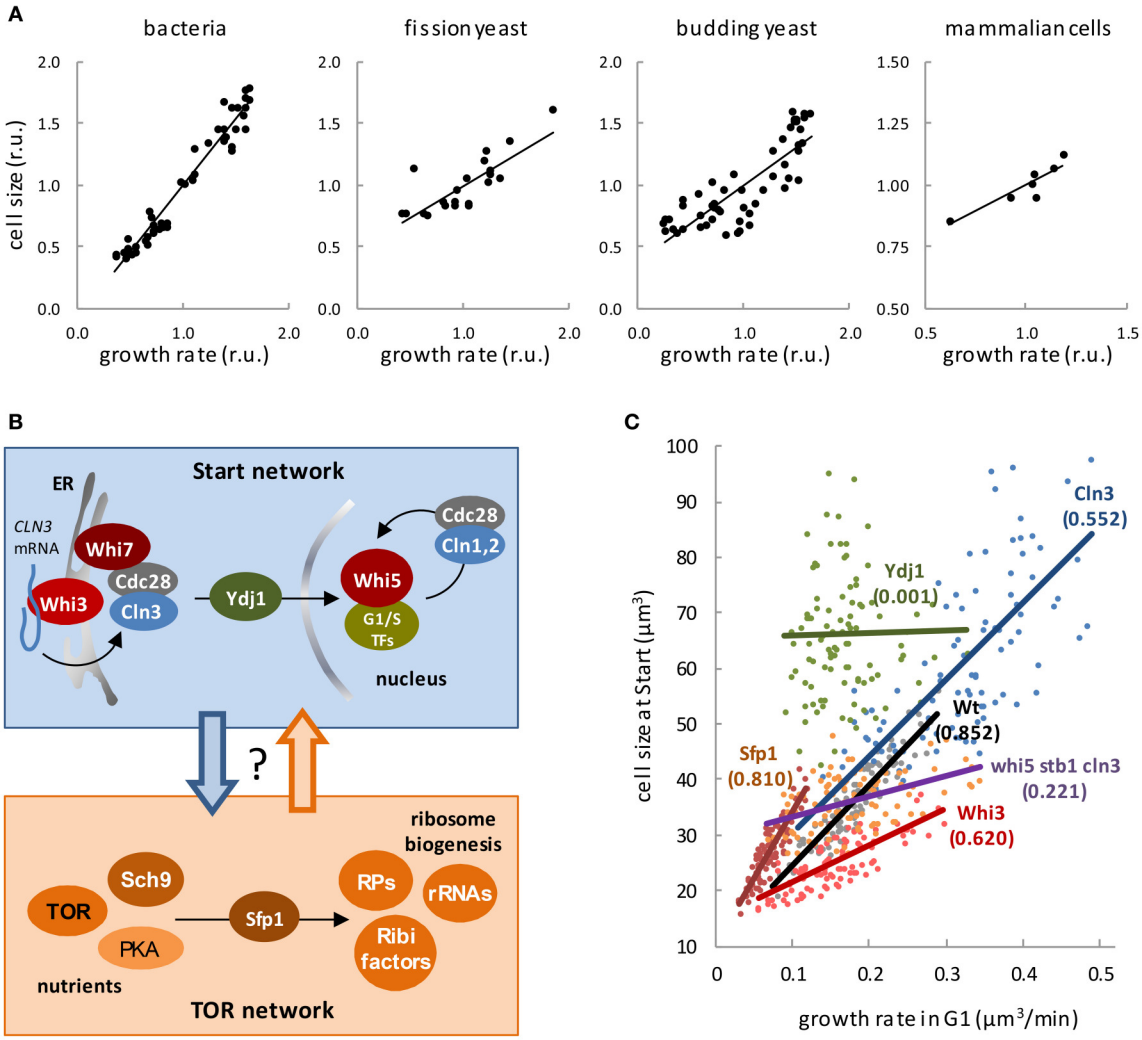
**Regulation of cell size by growth. (A)** Cell size as a function of growth rate in bacterial (Schaechter et al., 1958), fission yeast (Fantes and Nurse, 1977), budding yeast (Tyson et al., 1979), and mammalian (Hola and Riley, 1987) cells. **(B)** The Start and Tor networks in budding yeast. Top box. The most upstream activator of cell cycle entry, the G1 Cdk-cyclin complex (Cdc28-Cln3), phosphorylates Whi5 and induces the G1/S regulon. Additional cyclins Cln1, 2 ensure the G1/S transition by exerting a positive feed-back loop on transcriptional activation. Whi3 recruits Cdc28 and binds the *CLN3* mRNA to localize its translation and retain the Cdc28/Cln3 complex at the cytosolic face of the ER with the contribution of Whi7, thus preventing unscheduled cell cycle entry in early G1. Once cell size requirements have been met in late G1, Cln3 is released by specific chaperones as Ydj1. Bottom box. Nutrient and trophic factor signals are transmitted by different pathways to the TOR, PKA, and Sch9 kinases, which show complex reciprocal interactions. These central kinases activate ribosome biogenesis by inducing expression of ribosome biogenesis factors (Ribi), ribosomal proteins (RP) and rRNAs, which is mainly exerted through nuclear localization of transcription factor Sfp1. **(C)** Cell size at Start of wild-type budding yeasts cells and the indicated mutants as a function of growth rate in G1 (Ferrezuelo et al., 2012). Coefficients of correlation are indicated within brackets.

Unless stated otherwise, the term cell size is used here as an equivalent of cell volume, and growth rate as the inverse of the volume (or mass) doubling time. It is a classic observation that “bacterial cells increase in size during the lag which precedes cell division in a newly-inoculated culture, and become smaller again during the period of declining growth” (Henrici, 1928). Years later, Schaechter et al. carefully measured the size and macromolecular contents of *Salmonella typhimurium* cells growing in different media (Schaechter et al., 1958), and concluded that the size of these bacterial cells increased with growth rate. The same trend was also found in *Escherichia coli* (Pierucci, 1978) and in single-celled eukaryotes as fission (Fantes and Nurse, 1977), and budding (Johnston et al., 1979; Tyson et al., 1979) yeast, and diatoms (Von Dassow et al., 2006). Finally, similar effects on cell size have been observed in mammalian cells of different origins when analyzed under different trophic or nutritional conditions supporting different growth rates (Zetterberg et al., 1984; Zetterberg and Larsson, 1991; Rathmell et al., 2000; Conlon et al., 2001; Conlon and Raff, 2003; Dolznig et al., 2004), suggesting that cell size dependency on growth rate would be a universal property (Figure 1A). These data have been generally interpreted to support the idea that cells have specific mechanisms to modulate cell size as a function of nutrients or trophic factors. However, the same dependence of cell size on growth rate has been shown in individual yeast and mammalian cells displaying different growth rates under the same environmental conditions (Fantes, 1977; Hola and Riley, 1987; Ferrezuelo et al., 2012), which points to a more direct and deeper role of growth rate *per se* in the mechanisms that coordinate general biosynthetic processes and cell cycle progression. Supporting this notion, genetic manipulation of pathways that drive cell growth has a profound effect in cell size across the whole evolutionary scale as underlined in excellent reviews (Edgar, 2006; Cook and Tyers, 2007; Lempiäinen and Shore, 2009; Lloyd, 2013), and almost invariably with the same result: the faster the larger (Wertenbaker, 1923).

## Ribosome biogenesis as a general controller of growth rate and cell size

Ribosome biogenesis is the central target of the mechanisms that control cell growth from yeast to mammals (Arsham and Neufeld, 2006). In budding yeast, nutrients are sensed through the TOR, PKA, and Sch9 kinases (Figure 1B) to stimulate the nuclear localization of Sfp1, a transcription factor that drives expression of ribosomal proteins and ribosome biogenesis factors (Jorgensen et al., 2004; Marion et al., 2004). The first comprehensive screens for small cell mutants were performed in budding yeast (Jorgensen et al., 2002; Zhang et al., 2002). These studies underlined the relevance of ribosome biogenesis factors in cell size regulation, and showed that lower ribosome biogenesis rates due to poor nutrients or pathway malfunction cause a small cell size. However, reducing translation efficiency produces the opposite effect, i.e., a large cell size (Jorgensen et al., 2004). To reconcile these apparently conflicting observations, Jorgensen and Tyers (Jorgensen and Tyers, 2004) proposed that the rate of ribosome biogenesis, which correlates with nutrient quality, would somehow inhibit Start and force the cells to grow larger in G1. By contrast, a minimal translation rate would be needed to produce enough levels of G1 cyclins to activate Start (Schneider et al., 2004).

## Growth rate control on the start transition in budding yeast

Many components of the molecular regulatory network controlling Start (Figure 1B) have been involved in cell size control in budding yeast. The first identified small cell size mutant in yeast, *WHI1-1* (Sudbery et al., 1980), expressed a hyperactive version of Cln3 (Cross, 1988; Nash et al., 1988), a cyclin that acts with the Cdc28 kinase (Tyers et al., 1992, 1993) as the most upstream activator of Start (Johnson and Skotheim, 2013). Other proteins playing negative roles in the Start network were also identified by different screens for small cells and added to the *WHI* tale. Whi3 (Nash et al., 2001) is an RNA-binding protein that binds the *CLN3* mRNA and negatively regulates the Cdc28-Cln3 complex (Garí et al., 2001; Wang et al., 2004; Cai and Futcher, 2013; Holmes et al., 2013). Whi5 (Jorgensen et al., 2002) is a transcriptional inhibitor of the G1/S regulon (Costanzo et al., 2004; de Bruin et al., 2004) that is diluted by growth in G1 until a threshold is reached to execute Start, thus acting as a key sizer molecule in budding yeast (Schmoller et al., 2015). Finally, Whi7 is a Whi5 paralog that restrains nuclear accumulation of the Cdc28-Cln3 complex in G1 (Yahya et al., 2014). In summary, both ribosome biogenesis and the Start network are unequivocally involved in cell size regulation, but how essential are they to the outstanding dependence of cell size on growth rate?

In order to address this important question, a precise estimate of the growth rate in G1 and the cell volume at Start, the critical volume, was obtained from *individual* cells of a collection of strains lacking different factors of the Start network (Ferrezuelo et al., 2012). Cells lacking either negative (Whi3, Whi5) or positive (G1 cyclins, G1/S transcription factors) effectors showed lower degrees of correlation (Figure 1C). Of note, whereas the average cell volume was not grossly affected (Wang et al., 2009; Ferrezuelo et al., 2012), cells lacking simultaneously Cln3 as activator and Whi5 and Stb1 as inhibitors of Start failed to show a significant correlation between the critical volume and growth rate. By contrast, loss of Sfp1 caused a strong decrease in both growth rate and cell size at Start, but did not affect the correlation. Most remarkably, loss of one single protein, the Ydj1 chaperone, totally abolished the dependency of the critical volume on growth rate (Figure 1C). Ydj1 is involved in the degradation pathways of Cln3 (Yaglom et al., 1996), and plays a limiting role in nuclear accumulation of the Cdc28-Cln3 complex in late G1 (Vergés et al., 2007). On the other hand, Ydj1 is extensively involved in fundamental growth processes, suggesting that cell size control would lie at the interface between the cell cycle and cell growth machineries (Aldea et al., 2007). Although specific mechanisms exist that modulate components of the Start network as a function of nutrients (Baroni et al., 1994; Tokiwa et al., 1994), a general mechanism directly linking growth rate to the Start network would make cell size control more simple and comply with a single proposition: each cell must reach a critical size, which is set by its own growth rate, to execute Start. Thus, instead of having many pathways regulating the critical size as a function of each environmental factor involved, cells would gauge their own growth rate to integrate environmental information and modulate the critical size at a single-cell level. Indeed, ribosome biogenesis (Jorgensen and Tyers, 2004) could actively integrate all extrinsic and intrinsic signals to set growth rate and, as a result, cell size.

## The “speedometer” model of cell growth rate—critical cell size coupling

**Figure 2:**
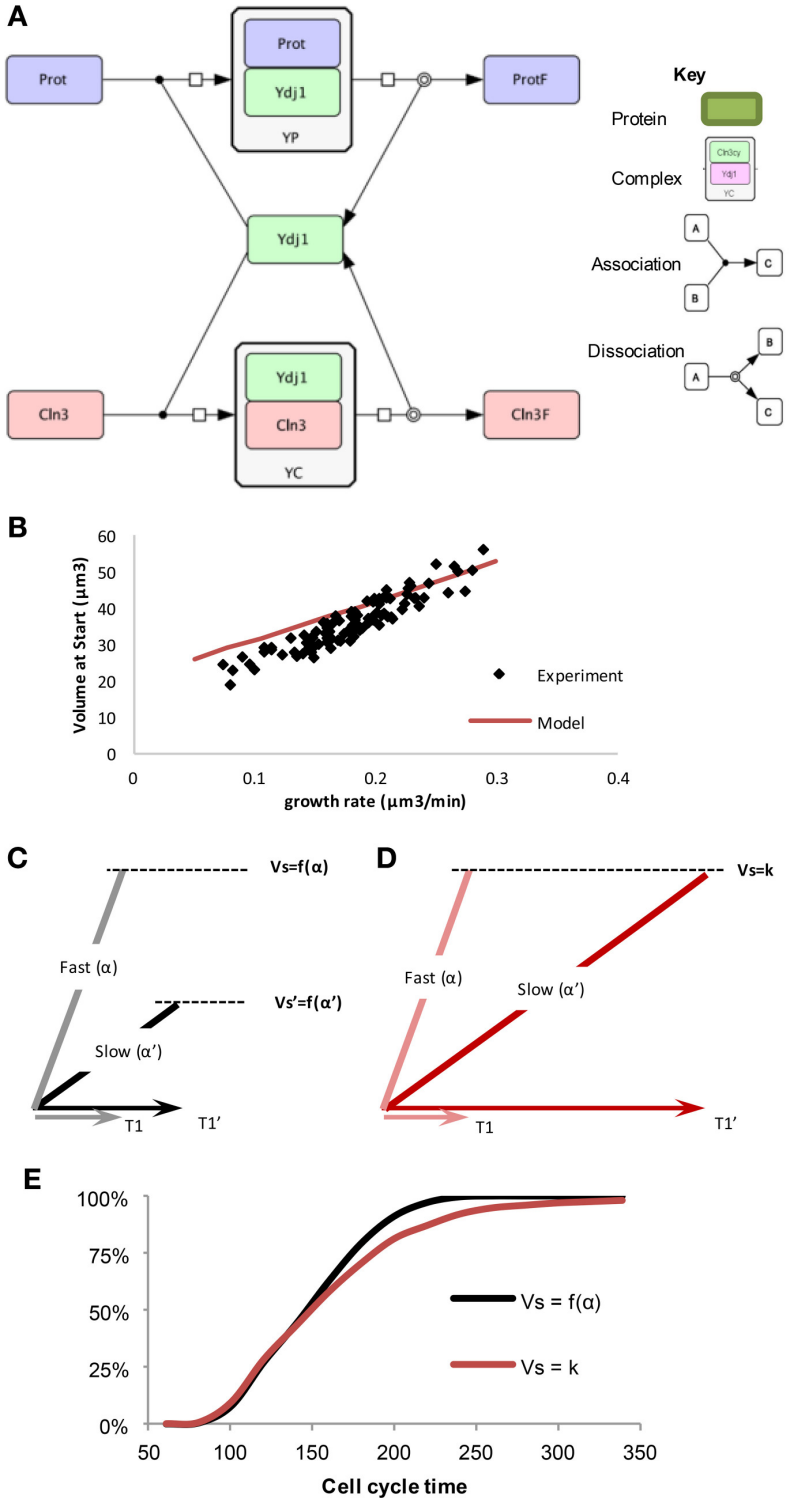
**The “speedometer” model of cell growth rate—critical cell size coupling. (A)** Competition between *prot* and *Cln3* for free *Ydj1*, drawn in CellDesigner (http://www.celldesigner.org/). **(B)** The “speedometer” model qualitatively predicts cell size as a function of growth rate. Experimental data as in Figure 1C. **(C,D)** Length of G1 (T1) have a larger noise and slow growing cells have much longer G1 in the model with fixed critical size, compared to G1 length in a growth rate dependent critical size model. **(E)** Cumulative distribution of cells finishing the cell cycle starting from a population of random newborn daughter cells in the two sizer models (Ferrezuelo et al., 2012).

As mentioned above, chaperone Ydj1 is the most important factor coupling the individual growth rates of cells to their critical size at Start. On the other hand, Ydj1 is the most abundant Hsp40 chaperone in budding yeast, binds hundreds of proteins (Gong et al., 2009) and is involved in key processes in cell growth (Caplan et al., 1992; McClellan et al., 1998). Finally, the same chaperone is responsible for the folding and release of Cln3 molecules to trigger Start (Vergés et al., 2007). The wiring diagram (Figure 2A) representing these molecular interactions can be turned into a mathematical model wherein *Cln3* and other Ydj1 client proteins (*prot*), compete for *Ydj1*. Both *Cln3* and *prot* need to bind to *Ydj1* to fold correctly, as indicated by the respective complexes (*YP* and *YC*), which produce folded cellular proteins (*protF*) and folded/free Cln3 (*Cln3f*). The rate of synthesis for *prot, Ydj1*, and *Cln3* depends on volume, and a threshold is set for the number of *Cln3f* molecules required in the nucleus to trigger Start. Protein (*prot*) synthesis determines how fast the cell grows. Thus, *prot* synthesis rate is assumed to correlate with growth rate and the model can be interrogated to predict the volume at which *Cln3f* level reaches the fixed critical value that is determined by Whi5 level. The model does not need to assume whether cells grow linearly or exponentially as it does not simulate the behavior as a function of time, and only considers the steady state solution for the dependence of volume at Start on growth rate. Indeed, the model predicts closely the observed correlation between growth rate and critical cell size (Figure 2B). Thus, the speedometer model of chaperone-driven communication of growth rate to the cell cycle machinery can explain the growth-rate dependence of the critical cell size.

## The benefits of a growth rate dependent size control

After showing that growth rate directly can determine the critical size of cells, the following question arises: What advantage would cells acquire by setting their size as a function of growth rate instead of having a fixed critical size?

To test these two scenarios, a model was created that could compare the different mechanisms for determining size: a fixed critical size and a growth-rate dependent size (Ferrezuelo et al., 2012). Experimental data shows that there is a distribution of different sizes of cells in a population when they enter Start. This could be due to two possible reasons. (1) There is a fixed critical size that, due to molecular noise (Di Talia et al., 2007), displays some stochasticity. (2) There is a distribution of possible critical sizes that are predetermined by the growth rate of each individual cell, such that slower growing cells have a smaller critical size than faster growing cells. Each cell in the population is initiated at a random size, according to the distribution of initial cell sizes in the experimental population. The cell then is randomly assigned a growth rate also based upon measured values. As the simulated cell grows, the size at which Start is initiated depends upon the method chosen.

Because of the forced fit to this data, the average cell size at Start will be the same in the simulations of both models, but length of G1 is longer on average in the fixed critical size model. This occurs because some of the slow growing cells spend a much longer time to reach the critical size and this is not compensated by more fast-growing cells dividing after a shorter time. Thus, slow growing cells display much shorter G1 periods comparing growth-rate dependent (Vs = f(α)) to independent (Vs = k) sizer mechanisms (Figures 2C,D). In other words, slow-growing cells must grow for a longer time if the critical size is not reduced as a function of growth rate. The resulting difference in the cumulative distribution of daughter cell-cycle times shows that after ~220 min all daughter cells in the growth-rate dependent critical size model would have divided at least once, while in the fixed critical size model it would have taken almost 350 min (Figure 2E). This difference clearly shows that a growth-rate dependent size control operating at a single-cell level could have evolved to optimize proliferation at the population level. Moreover, as slow-growing cells are more resistant to stress (Lu et al., 2009; Levy et al., 2012) and display a longer lifespan (Lin et al., 2000; Kaeberlein et al., 2005), a growth-rate dependent size control would provide the population with a significant evolutionary advantage.

If cell size had to be set by every single external environmental parameter, a myriad of signaling pathways to sense these conditions would be needed. However, in a growth-rate dependent model the sensor for nutrients and other external factors is incorporated into the growth rate, thus ensuring that the information is integrated within one intrinsic sensor, as opposed to many external sensors. This general model would be complemented by dedicated pathways to control cell size as a function of specific key nutrients (Baroni et al., 1994; Tokiwa et al., 1994; Gallego et al., 1997; Polymenis and Schmidt, 1997; Hall et al., 1998) or the metabolic status (Soma et al., 2014) of the cell, thus providing with a robust framework to adapt cell size to environmental conditions.

Based on our model, cell growth rate is communicated to the cell cycle through the level of free Ydj1 chaperone, which plays a crucial role in determining the rate at which Cln3 molecules are folded to trigger Start. Similar mechanisms could signal growth rate to other cellular processes that need to be scaled with size or to the growth potential of the cell.

## Author contributions

All authors listed, have made substantial, direct and intellectual contribution to the work, and approved it for publication.

### Conflict of interest statement

The authors declare that the research was conducted in the absence of any commercial or financial relationships that could be construed as a potential conflict of interest.
